# The distribution of Preputial vessels in different severity of rat congenital hypospadias model: imaging study using micro-computerized tomography

**DOI:** 10.1186/s12894-019-0547-4

**Published:** 2019-11-08

**Authors:** Defu Lin, Pei Liu, Guannan Wang, Weiping Zhang, Ning Sun

**Affiliations:** 1 National Center for Children’s Health, Beijing, China; 2 Department of Urology, Beijing Children’s Hospital affiliated to Capital Medical University, No.56 Nanlishilu Rd, West District, Beijing, China

**Keywords:** Prepuce, Blood vessel, Micro-CT, Hypospadias, Rat model

## Abstract

**Background:**

Micro-computerized tomography (micro-CT) is considered as an innovative non-invasive and high-resolution imaging technology. The current research aims to reconstruct the distribution of preputial vessels in different severity of rat congenital hypospadias model by micro-CT, and to provide an anatomic basis for the selection of preputial vessel pedicle flaps in surgery.

**Methods:**

Pregnant rats were exposed to finasteride from gestational day 12 to 17. Depending on the position of the urethral meatus, the pups were divided into normal, mild hypospadias and severe hypospadias groups. Six months after birth, the preputial blood vessels were observed in vascular perfusion with Microfil (a silicone-based polymer) and scanned by micro-CT. CTvox and NRecon were utilized to reconstruct 3-dimentional (3D) images. A pathological analysis of the specimen was taken in order to determine the position of Microfil.

**Results:**

The normal group and the mild hypospadias group had similar preputial image characteristics. At the junction of the inner and outer prepuce, the deep layer vessels of the superficial fascia were transversely distributed and formed a vascular ring-like structure. Among the severe hypospadias group, five had sufficient blood circulation while six had insufficient blood circulation. In sufficient blood circulation type, the ring-like vessels were found at the junction of the inner and outer prepuce similar to that of the normal and mild hypospadias group. However, only a small amount of capillary supply to this area in the insufficient type.

**Conclusion:**

The junction of the inner and outer prepuce with abundant blood circulation was suitable to be a vascular pedicle flap. The tubularized preputial island flaps were consistent with the ring-like vessels area, and the original blood supply was retained to the greatest extent.

## Background

The incidence of hypospadias is approximately 1:200~1:300 male births, which have doubled over the past three decades. The preputial skin is the principal grafting material to the urethroplasty [1, 2]. However, postoperative complications are about 5–25% [3], including urethral fistula, stricture and diverticula. The occurrences of urethral fistula and stricture are correlated to the blood supply of molding material for urethral reconstruction [4]. To create a more reasonable preputial vessel pedicle flap design to minimize the disruption of blood circulation, anatomical study is required to have a better understanding on the distribution of preputial vasculature.

The anatomic characteristic of the blood supply is difficult to detect, because the capillaries of prepuce are the terminal branch and measured in micron [5]. Micro-CT scanners reach spatial resolution in submicron level [6–9]. So far little research has been carried out in the study of preputial vascular distribution of patients with hypospadias. Previous literature has reviewed the anatomy of hypospadias preputial vessels using methods such as trans-illumination, microscopic observation and 3D computer reconstruction [10–12]. Although these approaches have studied the vascular anatomy, the preputial vascular networks are still difficult to measure in 3D.

Exposure to finasteride, estrogen and other anti-androgens in pregnant rat can induce congenital hypospadias model [13–16]. This is the first study identifying the distribution of the preputial vessels in rat hypospadias mode. The micro-CT, which was used extensively to detect the anatomy of the animal, was used to scan the rat hypospadias penile specimens to produce the 3D images of the preputial vessels [17].

It is hypothesized that the deep layer vessels of the superficial fascia were transversely distributed at the junction of the inner and outer prepuce. According to this distribution, a transvers preputial island flap was selected to preserve the blood supply to the greatest extent. The current research aims to reconstruct the distribution of preputial vessels in different severity of rat congenital hypospadias model by micro-CT, and to provide a theoretical basis for the selection of preputial vessel pedicle flaps in surgery.

## Methods

### Hypospadias rat model

The animal ethics committee’s approval was achieved by the Institutional Animal Care and Use Committee of Beijing Children’s Hospital, Capital Medical University prior to the experiment. Total 6 pregnant Wista rats (250-270 g) obtained from Animal Experimental Center (Capital Medical University, China) were housed in a temperature-controlled room in plastic cage (1 animal per cage) with free access to food and water at 22–25 °C on a 12 h light/dark cycle. The pregnant rats were divided into an experimental group (*n* = 4) and control group (*n* = 2). The experimental group received daily finasteride (Yuan Sen pharmaceutical Co. Ltd., Hebei, China) injection of 40 mg/kg to the subcutaneous of the abdominal wall, from 12 to 17 gestational days (GDs). The control group was injected with same volume of normal saline solution only. After delivery (GD22–24), mothers and the newborn rats were fed in one cage. On the 28th day after delivery, hypospadias rat models were selected from the neonatal rats. For testing purpose, rat models with the following features were used in the experiment: deficient foreskin ventrally with dorsal hood; abnormally located urethral meatus; vary degrees of ventral penile curvature.

### Silicone rubber compound infusion

Microfil infusion on rat models was started at the age of 6 months. Urethane was given to the rat models through intraperitoneal injection of 6 mL/kg to induce general anesthesia. The abdominal wall was opened using a midline incision. The abdominal aorta and the inferior vena cava were isolated and ligated. 24G indwelling needle was inserted to the abdominal aorta at the distal of the ligation point as an input channel. A 2 mm hole was cut at the inferior vena cava wall as an output channel. The micro-pump was connected (Product model: SDS-MP09, Shan De Shi medical company, Beijing, China), and 50 ml 40 °C heparin saline (50 U/ml) at 5 ml/min was input to dilate blood vessels and anticoagulation. Microfil (MV^− 122^ compound 4 ml, diluent solution10ml, curing agent 0.7 ml, Circulation Tech Company, America) 2 mL/min was added to replace heparin when the viscera turned pale. The perfusion was finished until the viscera became yellow. The specimens were stored at 25 °C to clot the Microfil, then the penile specimen was cut and stored in 4 °C environments. The specimens were saved in 10% formalin. After experiment, animals were deeply anesthetized by intraperitoneal injection of urethane to minimize the pain.

### Micro-CT scan

The Bruker SkyScan 1172 micro-CT system (Bruker, Kontich, Belgium) was utilized. Technical protocol of the Scanning was specified as follows: voltage of 59 kV and current of 167 μA with a 0.5-mm aluminum attenuation filter. A 6.88-μm resolution was obtained. Acquisition time for each specimen was 120 min. Raw projection image files were reconstructed using the Fledkamp cone-beam algorithm in NRecon Reconstruction and CTvox (Bruker) software [18].

### Pathological section

After image acquisition, the samples were embedded in paraffin and horizontally sectioned (7 mm of thickness). Samples were stained with hematoxylin-eosin (HE), rinsed in distilled water and mounted. Sections were assessed and the Microfil particles were detected using light microscopy.

## Results

### Establishment of hypospadias model

The control group rats gave birth to 24 fetuses, including 10 females and 14 males. All male offspring had normal penis without hypospadias. The experimental group gave birth to 41 fetuses, including 17 females and 24males. Totally, 16 hypospadias offspring were included. According to the location of the meatus, 16 hypospadias pups were further divided into mild group (*n* = 3) and severe group (*n* = 13).

### The morphology of the hypospadias model

**Fig. 1:**
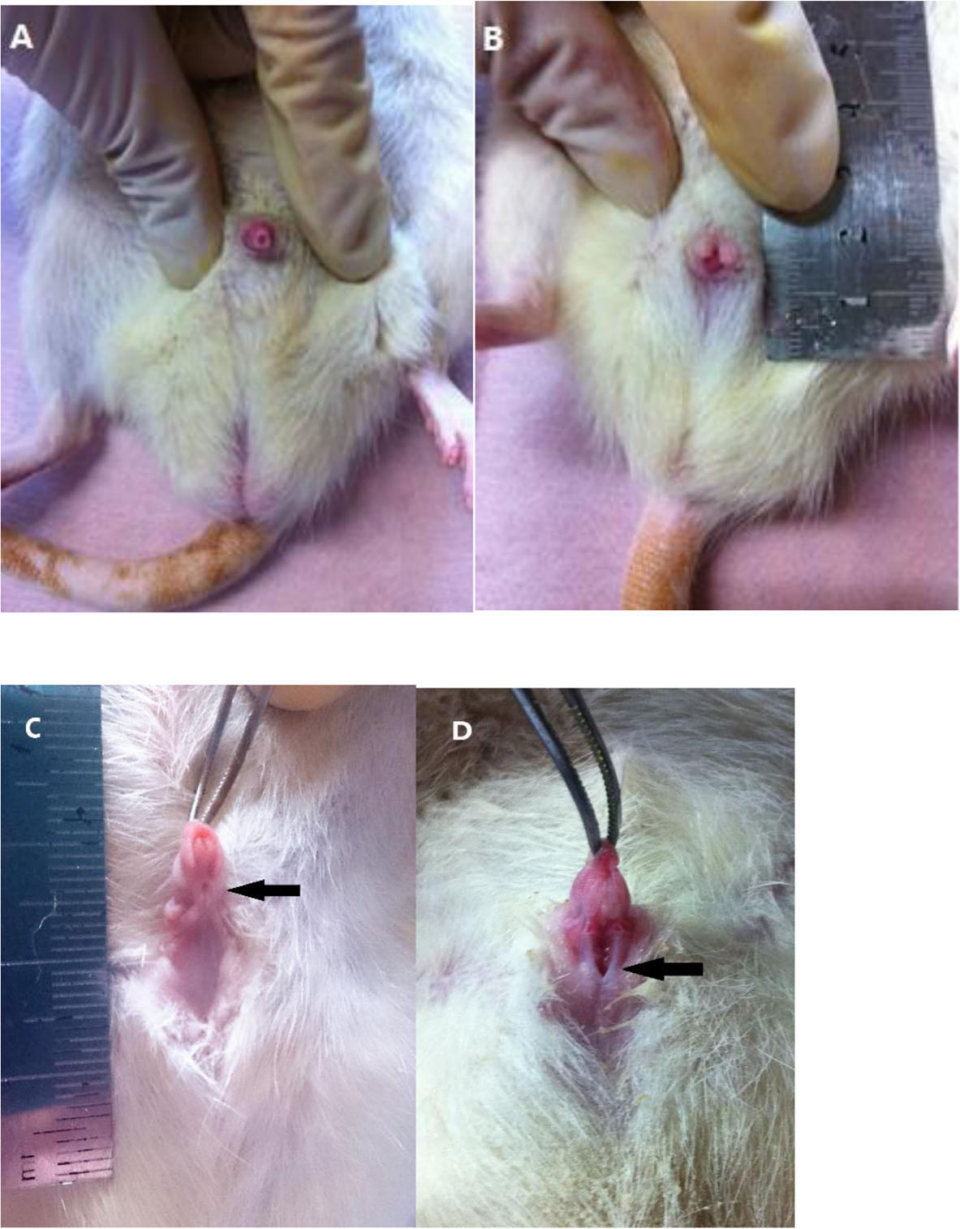
**a** The normal penis appearance. **b** The “V” shape prepuce of the hypospadias model. **c** Mild hypospadias model. **d** The severe hypospadias model. Arrows show the ectopic urethral meatus

In the control group (Fig. 1a), the urethral meatus are normally located. Prepuce covered the penis completely, and no ventral penile curvature was found. The urethral meatus was situated in the ventral of the ventral shaft in the mild hypospadias model (Fig. 1c). In the mild hypospadias group, there was ventral prepuce defect and a “V” shape dorsal prepuce (Fig. 1b). The glans was observed and the ventral penile curvature was not obvious in the normal position. The severe hypospadias model has urethral meatus located at the perineum. While the appearance of the prepuce and glans was similar between the severe and the mild hypospadias model, meatus fibrosis, which may induce penile ventral curvature, was observed on both sides of the ectopic urethral meatus in the severe hypospadias model (Fig. 1d).

### Penis images

**Fig. 2:**
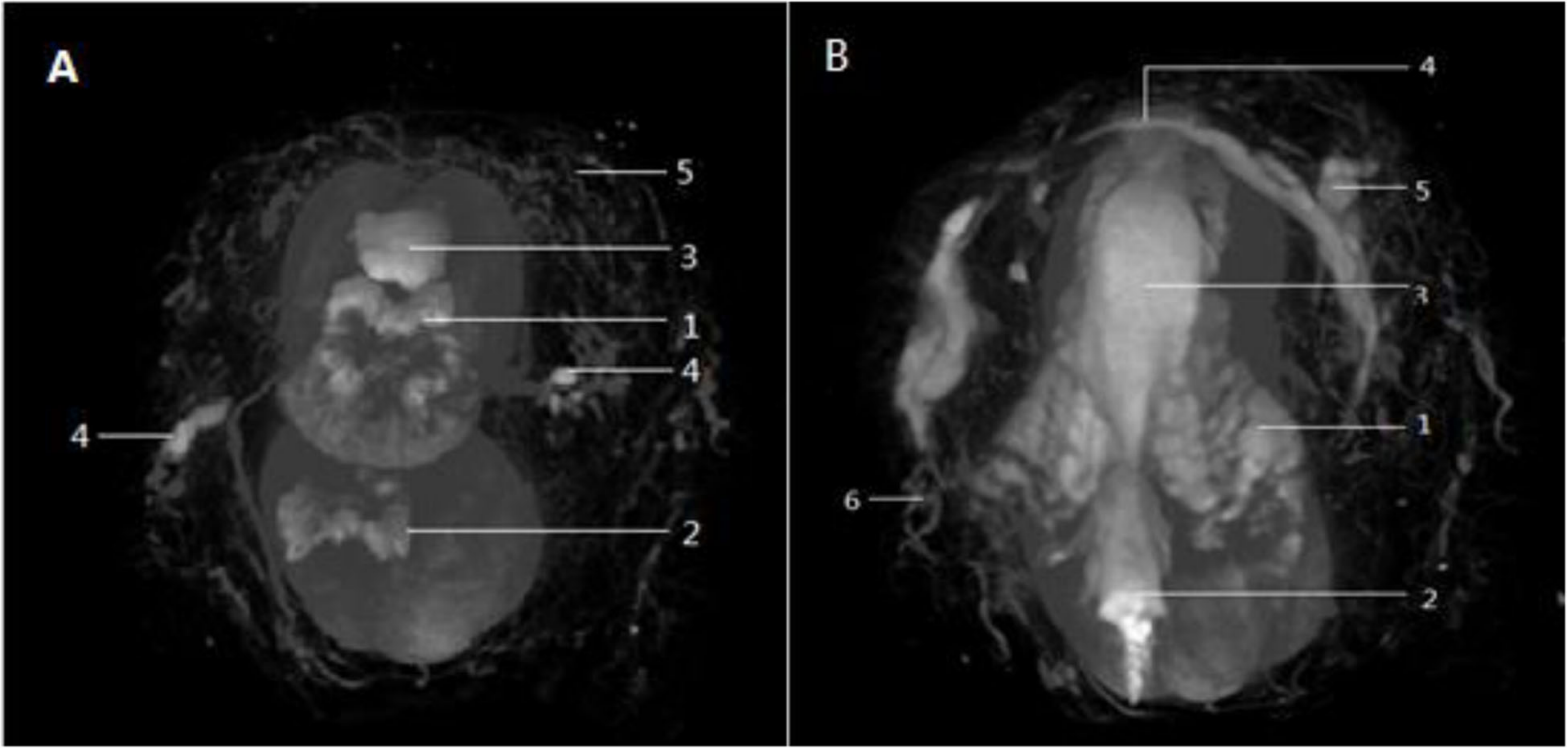
**a** Transverse section image at the level penile shaft showing (1) the bilateral corpora cavernosa connected with each other at the midline, (2) the corpus spongiosum was located on the ventral side and relatively smaller than the corpora cavernosa, (3) the dorsal penile vessels, (4) the two deep layer vessels of the superficial fascia were axially distributed at the lateral of the penile shaft in the area between 9- to 8-o’clock and 3- to 4-o’clock positions. (5) Innumerable superficial layer vessels of the superficial fascia were distributed at the surrounding of the penis to supply blood to the skin. **b** Transverse section image at the level of the glans showing (1) the corpora cavernosa separated by the (2) baculum, (3) showing the dorsal penile vessels. (4) The deep layer vessels showed a transverse distribution and formed a ‘ring-like structure’ at the junction of the inner and outer prepuce. The ‘ring-like structure’ was oriented from bilateral (5) the deep layer vessel of the superficial fascia

Tissues of different density were observed easily by adjusting the CT threshold value. The anatomy of penile and preputial dartos vessels, penile dorsal vessels, corpora cavernosa, corpus spongiosum, baculum was visualized clearly. (Fig. 2).

### Normal rat preputial blood vessels

**Fig. 3:**
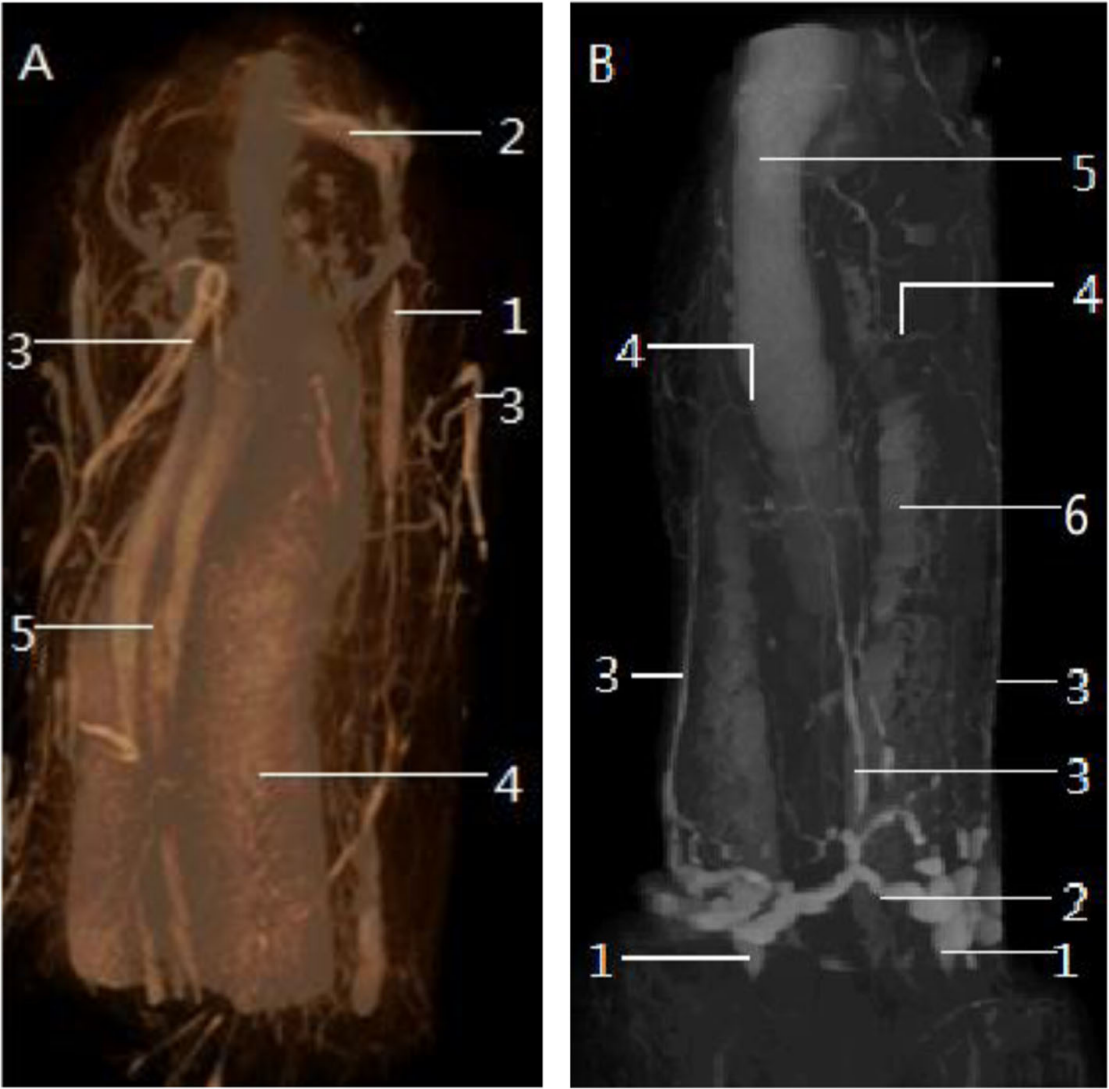
**a** The penis and preputial appearance in the nature position. (1) The deep layer of the penile dartos vessels go along with penile shaft at the dorsolateral position. At the junction of the inner and outer prepuce, the vessel transversely oriented terminal bifurcations anastomose with similar vessels of contralateral side and form a (2) vascular ring-like structure. (3) The superficial layer of the dartos vessels. (4) The corpora cavernosa, (5) the corpus spongiosum. **b** Reflecting the prepuce to the level of penopubic junction. (1) The bilateral deep layer of the penile dartos vessels converged and formed (2) the vascular ring, and then send (3) some parallel terminal blood vessels to the inner preputial dartos, these terminal blood vessels (4) anastomosis with each other. (5) The dorsal penile vessels. (6) The corpora cavernosa

The deep layer vessels of the superficial fascia, which was from the external pudendal artery, were axially distributed bilaterally. At the junction of the inner and outer prepuce, bilateral deep layer vessels showed a transverse distribution and form a vascular ring-like structure. The ring-like structure continued to send parallel terminal vessels to the inner preputial skin (Fig. 3b). These terminal vessels formed numerous reticular lateral branches. The superficial layer vessels of the superficial fascia supplied blood to the penile skin. The deep and superficial layers vessels can be observed clearly by adjusting the viewing angle in 3D. There was no noticeable relationship between the superficial and deep vessels of prepuce. The diameter of the superficial vessel was thinner than the deep one, but the superficial one had a greater number of blood vessels and branches (Fig. 3).

### Hypospadias model preputial blood vessels

**Fig. 4:**
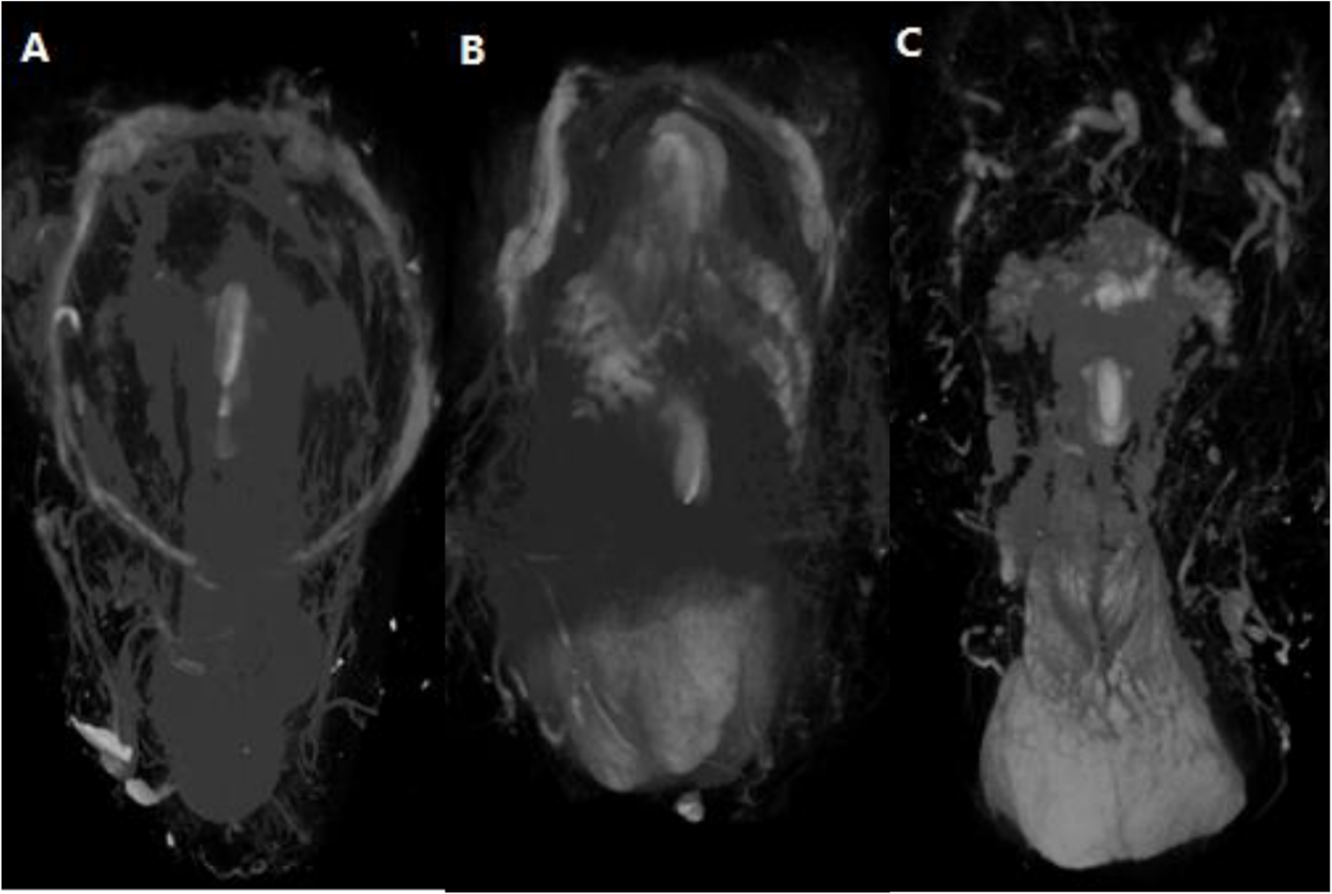
The 3D images reconstructed by the micro-CT.(A) Mild hypospadias. (B) Severe hypospadias with good blood circulation. (C) Severe hypospadias with poor blood circulation. **a** The terminal branches originated from the deep layer of the penile dartos vessels formed an intact vascular ring and covered the glans. **b** Because of the ventral part prepuce defected, the deep layer vessels converge to form a half ring structure at the dorsal of the glans. **c** The severe hypospadias with insufficient blood circulation, which was absent of dominant vessels, the vascular ring was replaced by a network structure

The distribution and morphology of the deep and the superficial layer vessels were similar between mild hypospadias model and normal penis. The bilateral deep layer vessels were converged to form an intact vascular ring at the junction of the inner and outer prepuce (Fig. 4a).

The severe hypospadias group was divided into two subgroups based on their blood circulation of the deep layer vessels at the junction of inner and outer prepuce. In the good blood circulation group as showing in Fig. 4b, the deep layer vessels converged to form a half ring structure at the dorsal of the prepuce regardless of the fact that there was an absent of ventral prepuce. However, the vascular ring was replaced by a network distribution in the poor circulation group (Fig. 4c).

### Pathology

Microfil particles can be found in the arteries, capillary networks and veins. The diameter of Microfil was less than capillaries, so Microfil can fill the entire vascular system. No Microfil leakage was observed outside the blood vessel wall to the surrounding tissue, hence Microfil was an ideal angiography reagent.

## Discussion

Hypospadias is among the most common congenital defects in pediatric urology. Due to anterior urethral maldevelopment, the ectopic urethral meatus may be located anywhere from the tip of glans to perineum. Urethroplasty is the only method to treat hypospadias. There are more than 300 surgical techniques to treat this disease, but every technique has some corresponding surgical complications, which are most likely in urethral fistula. So far, there are no common standard surgical techniques have been approved by all pediatric urologists [19–21]. To figure out this problem, we analyzed the preputial vessels in different severity of the rat hypospadias model by micro-CT. It is generally believed that blood vessels in the prepuce have two layers which can be easily separated [10]. The junction of the inner and outer prepuce has the most abundant blood supply from the deep layer vessels and can serve as a preputial vessel flap. Innumerable superficial layer vessels are distributed at the surrounding of the penis to supply blood to the skin. This type of vascular distribution is the anatomic basis of tubularized preputial island flaps procedure, as it not only ensures blood supply to the flap, but also prevents penis skin necrosis, which has been demonstrated once again in this study.

The vascular ring structure was originated from the deep layer vessels of the superficial fascia. Four main types of the deep layer vessels had been described in human: single branch predominant type (41%), two branches predominant type (25%), arching H-type (12.5%), or net-like type (21%). The qualities of the vascular pedicle and the urethral-plate were the two important surgical variables [22].

Tubularized preputial island flaps are the ideal single-stage repairs for the proximal hypospadias. For patients with severe hypospadias and ventral curvature, two-stage operation is still meaningful [23]. Clinical data indicates that penile curvature corrected in the first phase. Urethroplasty is done in the second phase to reduce the incidence of complications. Two-stage surgery reduces the difficulty of the operation somewhat, on the other hand it increases surgical operation time and prolonged treatment time [24]. This experiment verified that in the normal group and the mild hypospadias group, the blood vessels at the junction of the inner and outer prepuce had a wider caliber and formed a vascular ring. While in the group with severe hypospadias, the blood vessels at the junction of the inner and outer prepuce had a relatively smaller caliber displayed a net-like distribution and could not constitute a clear and complete vascular ring. These results may explain patients with severe hypospadias are most likely to suffer from postoperative complications than the ones with mild hypospadias.

We divided the severe hypospadias group into two types based on their vascular morphology: The first type has a sufficient blood supply. Although the blood vessels at the junction of the inner and outer prepuce could not form a vascular ring completely due to the ventral prepuce defect, there was a semi-circular vessel structure, which diverges small branches into the inner preputial skin to ensure an efficient supply of blood; For the second type, there was no semi-vascular ring to support the efficient blood circulation. The vessels were netlike distributed and contained mainly capillaries so there was no sufficient supply of blood to the preputial vessel pedicle flap. This result may explain the fact that patients with severe hypospadias achieved satisfactory results without postoperative complications after one stage surgery because their blood vessels at the junction of inner and outer prepuce provided a sufficient amount of blood for a better survival of the flap. At the same time, we concluded that patients with severe hypospadias with poor vessels structures were highly recommended to undergo two-stage surgery to reduce postoperative complications.

The prepuce vessels are the terminal branch, and the capillaries are extremely narrow, hence knowledge about the vessels is limited. Considerable work has been done to study prepuce vessels, such as transillumination with endoscopic cold light source, microscopic observation of gelation and India ink perfusion, 3D reconstruction of histological sections [10–12]. In this study, an advanced technology, micro-CT scanning, was used to analyze the distribution of preputial vessels. Micro-CT is an innovative non-invasive and high-resolution imaging technology. O’Neill et al. found the results of virtual sectioning at micro-CT and conventional histologic sectioning are highly correlated [18]. The CTvox (Bruker) software can be utilized to observe the samples by transverse, sagittal, and coronal section image, and established 2- or 3-dimensional reconstruction on interesting parts. The highest resolution can reach 0.5 μm, while the mean diameter of capillary vessels is 6 to 9 μm, so micro-CT can be used to micro vessels scanning [25].

The limitation of this study is that human penile anatomy is different from penile anatomy of rats. This study may not represent such anatomy of the humans. The next step, the clinical experiment will be carried out to detect the exact relationship between the postoperative complication and the anatomy of the preputial vessels.

## Conclusion

The application of Microfil and contrasted micro-CT scanning improve our understanding on the anatomy of the hypospadias, especially the preputial vascular structure in 3D. The junction of the inner and outer prepuce with abundant blood circulation would be a suitable vessel pedicle flap. The tubularized preputial island flaps were consistent with the ring-like vessels area, and the original blood supply was retained to the greatest extent.

## Data Availability

The datasets generated and analyzed during the current study are available from the corresponding author on reasonable request.

## Abbreviations

3D: 3-dimentional
GDs: Gestational days
HE: Hematoxylin-eosin
Micro-CT: Micro-computerized tomography
